# Outcomes and complications of cerebral aneurysms operated on by eyebrow incision according to aneurysm type and location

**DOI:** 10.1186/s12893-023-01942-7

**Published:** 2023-03-08

**Authors:** Jin Eun, Ik Seong Park

**Affiliations:** 1 Department of Neurosurgery, Eunpyeong St. Mary’s Hospital, College of Medicine, The Catholic University of Korea, Seoul, Republic of Korea; 2 Department of Neurosurgery, Bucheon St. Mary’s Hospital, College of Medicine, The Catholic University of Korea, Bucheon-Si, Republic of Korea

**Keywords:** Trans eyebrow incision, Keyhole surgery, Aneurysmal neck clipping

## Abstract

**Objective:**

Trans-eyebrow supraorbital aneurysmal neck clipping, also known as keyhole surgery, have many advantages of minimal invasive surgery. However, there are few studies on whether there is a difference in keyhole surgery according to the location of the aneurysm, and how the complications after keyhole approach differ from the conventional approach. The authors investigated the surgical outcome of keyhole aneurysmal surgery for clarify the characteristics of keyhole surgery.

**Methods:**

A retrospective study was performed with review of medical records and images of patients with anterior circulation aneurysm undergoing aneurysmal clipping with keyhole surgery. The patient's clinical condition, imaging, surgical condition, and outcome were investigated.

**Results:**

As a result of analysis about the location of the aneurysm, middle cerebral artery (MCA) aneurysm group had a longer operation time than internal carotid artery and anterior cerebral artery aneurysm groups, but there was no significant difference in complication rate. The olfactory dysfunction occurred more than that of conventional surgery and occurred less in MCA aneurysm group than others. Scalp sensory change in the surgical site was more common in patients with unruptured aneurysms.

**Conclusion:**

By accurately investigating the frequency and severity of complications associated with trans-eyebrow aneurysmal neck clipping surgery, it can help to select a surgical approach considering risk versus benefit. In addition, patient’s satisfaction can be increased by providing information to patients and caregivers in advance about the outcome of this approach and the anticipated complications.

## Introduction

Trans-eyebrow supraorbital aneurysmal neck clipping is a typical example of minimal invasive surgery in which tissue damage is less likely to occur. Faster recovery and the patient's burden reduced [1–3]. However, it is not easy for beginners of aneurysmal clipping to choose this approach due to the prediction that the corridor is narrow, which limits the use of instruments and worries about narrow surgical field of view. Recently, there have been many reports about surgical outcomes of keyhole aneurysm surgery, but most of them focus on unruptured cases. There is little report which include large volume of ruptured aneurysms. And there are only few studies on how different complications are occurred compared to conventional surgery and how outcomes and complications differ depending on the location of aneurysms. The authors conducted a retrospective study to examine the difference of surgical outcomes between ruptured aneurysms and unruptured aneurysms and how keyhole surgery outcomes differ depending on the location of the aneurysm through more than 400 cases.

## Materials and methods

**Fig. 1:**
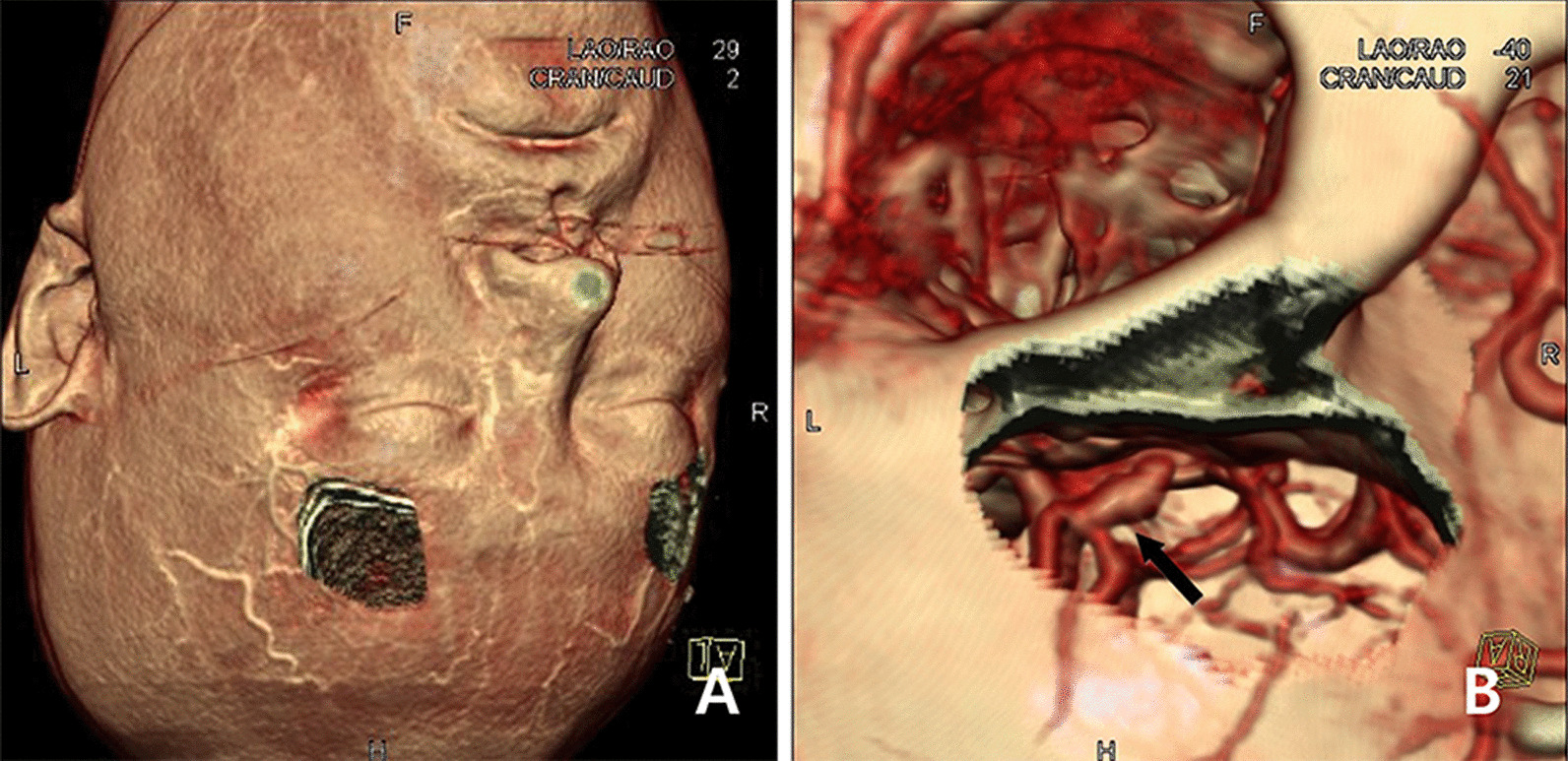
Three dimensional (3D) computed tomography (CT) images which was made during the surgical simulation at CT workstation. **A** A small opening was made on eyebrow area like surgical keyhole craniotomy with three dimensional surface rendering image. **B** After magnification and tilting of the image, anterior communicating artery aneurysm dome and neck were noticed through small opening (black arrow)

This is a retrospective study of 205 unruptured and 216 ruptured aneurysm cases undergoing keyhole clipping by a single neurosurgeon from July 2007 to September 2019 at a single institution. The authors evaluated the patient's neurological status at admission before surgery. Presurgical planning was conducted at computed tomography (CT) workstation according to the finding of three dimensional (3D) images which was made by SOMATOM Definition AS® 64 CT Scanner and Syngo 3D software (Siemens, Erlangen, Germany). A 2 × 2.5 cm size opening was made on the eyebrow area of the 3D CT image and surgical simulation was performed to confirm that the neck of the aneurysm was well observed through the supraorbital view (Fig. 1). Most of the patients with unruptured anterior circulation aneurysm selected as a candidate of keyhole surgery, but thrombosed or giant aneurysm were treated by the conventional craniotomy. In case of ruptured aneurysms, neurologically good condition (Hunt Hess grade 1,2) was given priority to keyhole surgery and neurologically poor grade patients (Hunt Hess grade 3 or higher) but with minimal brain swelling on brain CT image or neurologically deteriorated due to hydrocephalus were selected as a candidate of keyhole surgery.

In the analysis, patients’ neurologic conditions were assessed according to Hunt-Hess grade and Glasgow coma scale (GCS) before and after the surgery. The shape of aneurysm classified with 3 groups: saccular, elongated and others: Saccular aneurysm means aneurysm width and length ratio was within 1: 2. When the ratio was higher than 1:2, it was categorized as elongated aneurysm. Fusiform or complex shape aneurysms are classified as others. The total operation time was used as a representation for evaluating the difficulty of the operation. The operation time was defined as skin incision to skin closure time. To determine whether the aneurysm location is a limitation of keyhole surgery, the locations of aneurysms were divided into 3 groups and evaluated whether the outcome and complication of the operation were different. In detail, A1 and anterior communicating artery aneurysm classified as anterior cerebral artery (ACA) group, supraclinoid segment of the internal carotid artery (ICA) aneurysm as ICA group, M1 and middle cerebral artery (MCA) bifurcation aneurysm as MCA group. Short term post-operative complications that could affect the length of stay, such as meningitis, hygroma, subdural or epidural hemorrhage, infarction, intracerebral hemorrhage (ICH), and wound infection were investigated. After discharge, the patients were evaluated with patient questionnaire of health status at the outpatient clinic. We evaluated whether memory loss, olfactory dysfunction, and scalp sensory change which are thought to have an impact on the patient's quality of life in the future, although not directly affecting the length of hospital stay happened or not. For each patient group, follow-up evaluation was performed at the outpatient clinic at 6 months after the operation, and it was checked whether there was any change affecting daily life compared to before surgery.

### Surgical technique

All the surgery performed under general anesthesia. The head was positioned slightly vertex down on the horseshoe without pinning. The degree of vertex down was adjusted to case by case according to the CT simulation performed before surgery.

**Fig. 2:**
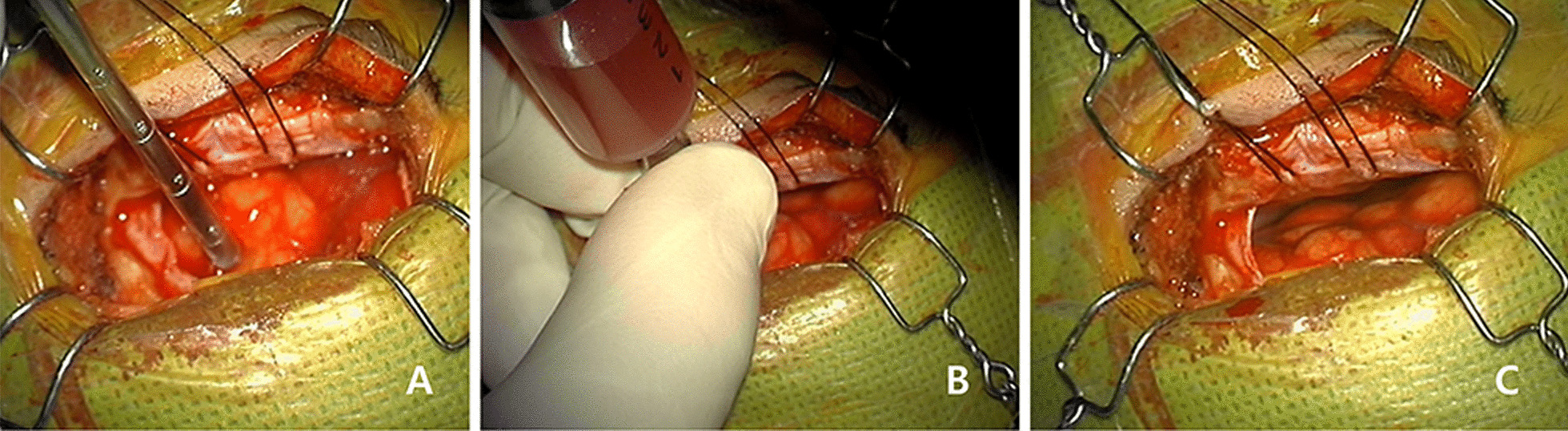
Ventriculostomy procedure during ruptured aneurysmal clipping by keyhole surgery. **A** Ventricular access was conducted by the perpendicular puncture of brain tapp needle. There was no room between the dura and brain surface due to brain swelling. **B** A total of 40 ~ 60 ml of cerebrospinal fluid (CSF) was drained by the repeated aspiration with syringe. **C** After CSF drainage, brain relaxation was achieved easily

Eyebrow shaving was not performed, and only the alcohol sponge was used for skin disinfection before drape. Skin incision was made on the center of eyebrow. Supraorbital nerve damage was minimized by dissecting supraorbital nerve up to 3 cm from the supraorbital notch, avoiding incision beyond the lateral margin of the eyebrow can prevent damage to the frontal branch of the facial nerve. A small burr hole was made in a superior temporal line using a ball type drill bit, and a 2 × 3 cm free bone flap was made with preserving a superior orbital rim close to the anterior skull base. Craniotomy was performed using a pediatric electrical saw to minimized bone defects as much as possible. In order to expand the surgical field of view, before opening the dura mater, the inner edge of the skull was drilled out to minimize brain retraction. The dura mater was incised in a semi-circular shape and reflected to the outside. Gentle retraction of orbitofrontal gyrus provides some room for cerebrospinal fluid (CSF) drain with suction device and a gradual drain provide a direct view of the arachnoid membrane of chiasmatic and carotid cistern. When the cisterns were opened, CSF suction proceeded quickly to facilitate proximal internal carotid artery (ICA) exposure and proximal control. In ruptured aneurysm cases, arachnoid dissection and CSF drain may not be available due to subarachnoid hemorrhage and brain swelling. In these situations, CSF drainage through ventriculostomy aspiration of 40 to 60 ml of CSF provided an adequate view around the carotid cistern without frontal lobe retraction (Fig. 2). Dissection between the frontal lobe and the optic nerve was performed and the laminar terminalis behind the optic chiasm was exposed. Since then, operation was done in the same way as conventional aneurysmal clipping. Detailed surgical procedures refer to our previous report [4].

### Statistical analysis

The software used for statistical analysis was R (ver 3.6.2). Each variable was analyzed in available patients after censoring missing data. For descriptive statistics of data, median with interquartile range (IQR) and frequency counts with percent were used. A univariable analysis was conducted to see the effect on the surgical outcome of each variable. For testing the significance of the continuous variables (age and aneurysm size) in operation time and event rates, linear model and logistic regression model were set and the significance of the parameters of variables were tested by F test and likelihood ratio test, respectively. When building a linear model, spline method was considered because linearity assumption of model was not satisfied between predictor and response. For other non-continuous (grouped) variables, the mean operation time was compared by Welch’s t-test or ANOVA between groups, and the significance of differences in the event frequencies was tested through Pearson's chi-square test, Fisher’s exact test in case of low frequency counts, and Cochran-Mantel–Haenszel statistics for ordinal variable such as GCS group. A multivariable analysis was also performed in which several independent variables were considered at the same time. As in the case of the univariable analysis, linear model and logistic regression model were used for analysis of operation time and the other events respectively, and spline method was also considered for a continuous variable. For each independent variable, decision whether to include in the model or not was based on the significance of the parameter of each variable (p < 0.10), and stepwise algorithm was used. Interaction effects between variables were also considered last. All p values presented in this paper were two-sided.

## Results

### Baseline characteristics

We analyzed the difference depending on the location of the aneurysm and whether it was ruptured (Table 1). There was no difference in age distribution according to location, and female patients were more in all three groups, and the difference was the lowest in ACA aneurysm group. In ICA aneurysm group, the ratio of unruptured and ruptured aneurysm was similar as 47.2%: 52.8%, but there were more ruptured aneurysm cases in ACA aneurysm group and more unruptured aneurysm cases in MCA aneurysm group (p < 0.001). Aneurysm configuration was usually saccular type in all three groups (p = 0.015). GCS, aneurysm size and Intraoperative rupture (IOR) did not show different distribution in the three groups (p = 0.712, p = 0.854, and p = 0.996, respectively).

**Table 1:** Baseline characteristics according to location classification

Variable	By location			P-value
	ICA aneurysm	ACA aneurysm	MCA aneurysm	
*Age (Nav)*	106	174	141	
Median (1Q-3Q)	54 (46–64)	52 (45–61)	55 (47–64)	0.222^a^
*Sex (Nav)*	106	174	141	0.002^b^
Male (%)	22 (20.8)	71 (40.8)	46 (32.6)	
Female (%)	84 (79.2)	103 (59.2)	95 (67.4)	
*Rupture (Nav)*	106	174	141	< 0.001^b^
Unruptured (%)	50 (47.2)	65 (37.4)	88 (62.4)	
Ruptured (%)	56 (52.8)	109 (62.6)	53 (37.6)	
*GCS (Nav)*	105	173	141	0.712^c^
Good (13–15)	94 (89.5)	157 (90.8)	131 (92.9)	
Fair (9–12)	6 (5.7)	10 (5.8)	5 (3.5)	
Poor (3–8)	5 (4.8)	6 (3.5)	5 (3.5)	
*Aneurysm size (Nav)*	98	161	131	
Median (1Q-3Q)	5.00 (4.00 – 7.00)	5.00 (3.00–6.00)	5.00 (3.66–6.00)	0.854^a^
*Aneurysm configuration (Nav)*	85	140	116	0.015^b^
Saccular (%)	64 (75.3)	99 (70.7)	88 (75.9)	
Elongated (%)	14 (16.5)	10 (7.1)	8 (6.9)	
Others (%)	7 (8.2)	31 (22.1)	20 (17.2)	
*IOR (Nav)*	106	174	140	0.996^b^
No (%)	99 (93.4)	163 (93.7)	131 (93.6)	
Yes (%)	7 (6.6)	11 (6.3)	9 (6.4)	

*ACA* anterior cerebral artery, *GCS* Glasgow coma scale, *ICA* internal carotid artery, *IOR* intraoperative rupture, *MCA* middle cerebral artery, *Nav* number available

^a^ Kruskal–Wallis rank sum test

^b^ Pearson’s Chi-squared test

^c^ Cochran-Manel-Haenszel test scoring each GCS group as its median GCS

In comparison according to ruptured status, there was no significant difference in gender (p = 0.176). Median age of unruptured aneurysm group was higher than ruptured group, 56 vs. 51 years old (p = 0.005). The distribution of the aneurysm configuration was not different, and the median size of the ruptured aneurysm was 5.00 mm, which was significantly higher than that of the unruptured aneurysm, 4.46 mm (p < 0.001). The occurrence of IOR was found to be significantly higher in ruptured aneurysm (p = 0.003) (Table 2).

**Table 2:** Demography according to ruptured status

	By ruptured status		P-value
	Unruptured	Ruptured	
*Age (Nav)*	203	218	0.005^a^
Median (1Q-3Q)	56 (48–64)	51 (45–61)	
*Sex (Nav)*	203	218	0.176^b^
Male (%)	60 (29.6)	79 (36.2)	
Female (%)	143 (70.4)	139 (63.8)	
*Aneurysm configuration (Nav)*	175	166	0.227^b^
Saccular (%)	126 (72)	125 (75.3)	
Elongated (%)	21 (12)	11 (6.6)	
Others (%)	28 (16)	30 (18.1)	
*Aneurysm size (Nav)*	192	198	< 0.001^a^
Median (1Q-3Q)	4.46 (4.00–5.00)	5.00 (4.00–7.00)	
*Median (1Q-3Q) (%)*	4 (3–5)	5 (4–7)	
*IOR (Nav)*	203	217	0.003^b^
No (%)	198 (97.5)	195 (89.9)	
Yes (%)	5 (2.5)	22 (10.1)	

*IOR* intraoperative rupture, *Nav* number available

^a^ Wilcoxon rank sum test

^b^ Pearson’s Chi-squared test

### Analysis of operation time

**Fig. 3:**
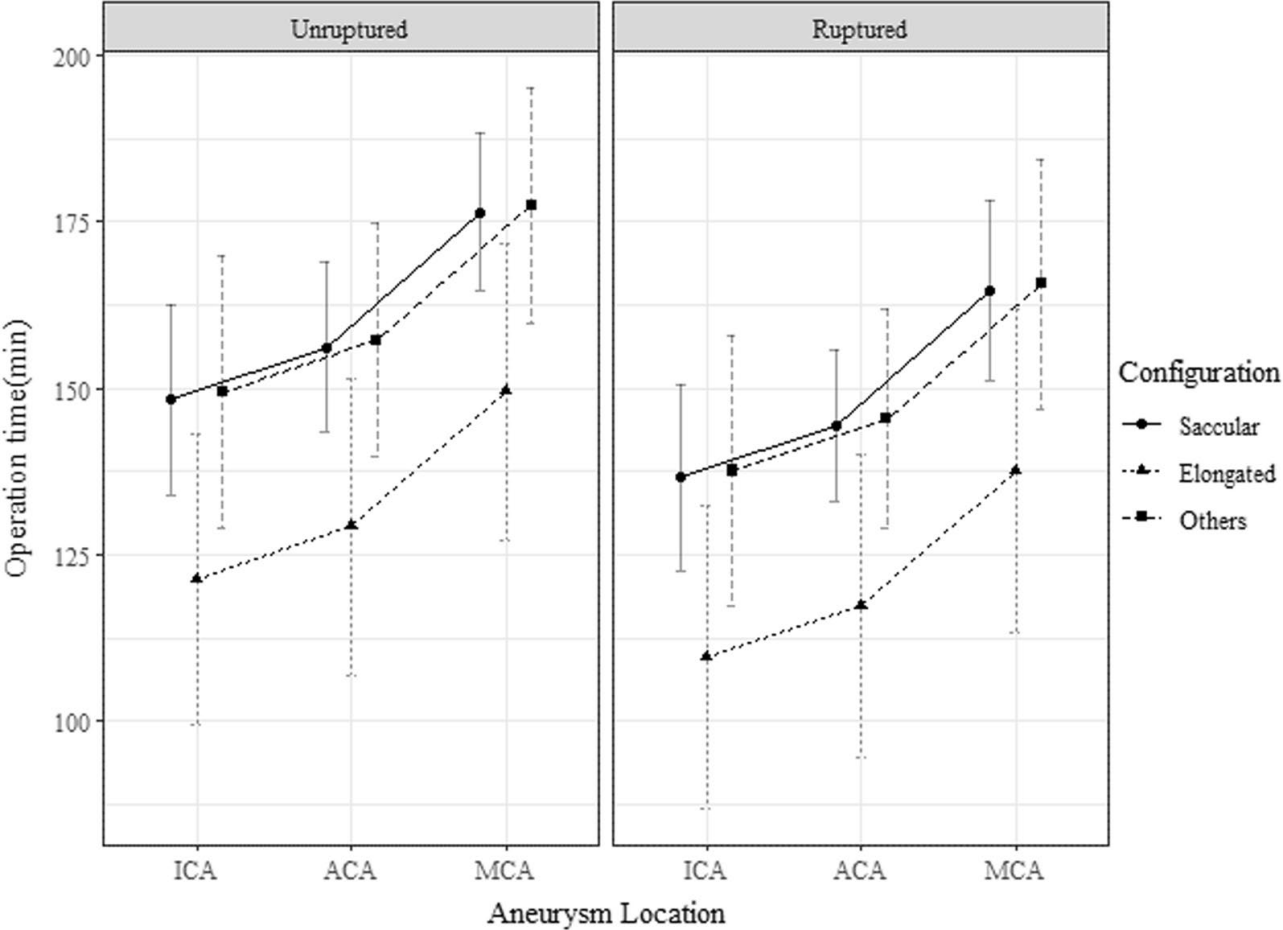
Operation time by ruptured status, configuration and locations. *ACA* anterior cerebral artery, *ICA* internal carotid artery, *MCA* middle cerebral artery

In univariable analysis for the operation time, the unruptured aneurysm group was found to have a longer operation time than the ruptured aneurysm group (p = 0.023). Clinical grade, aneurysm size, aneurysm location, and aneurysm configuration also affect significant differences in operation time (p = 0.041, p = 0.031, p < 0.001, p < 0.001, respectively) (Table 3). Multivariable model showed aneurysm location and configuration have significant effect on operation time, and whether aneurysm was ruptured or not showed borderline significance (p = 0.002, p = 0.043, p = 0.064, respectively). In post-hoc analysis with Bonferroni p value correction for location and configuration, MCA aneurysm group had significantly longer operation time than other locations (adjusted p: vs. ICA aneurysm = 0.002/ vs. ACA aneurysm = 0.018) and the elongated aneurysm group tended to have a shorter operation time but the adjusted p value showed a borderline significance comparing with non-saccular or fusiform type aneurysm group (adjusted p: vs. Saccular configuration = 0.041 / vs. Other type configurations = 0.090) (Fig. 3).

**Table 3:** Analysis of operation time

Variable (Nav)	Operation time (Mean, SD)	P-value
Total (421)	154 (58)	
*Rupture*		0.023^a^
Unruptured (203)	161 (66)	
Ruptured (218)	148 (49)	
*Hunt-hess grade*		0.041^b^
Grade 0 (202)	160 (66)	
Grade 1 – 2 (161)	145 (50)	
Grade 3 – 5 (58)	156 (48)	
***Aneurysm size (390)***	153 (59)	0.031^c^
*Location*		< 0.001^b^
ICA aneurysm (106)	142 (54)	
ACA aneurysm (174)	149 (56)	
MCA aneurysm (141)	169 (62)	
*Aneurysm configuration*		< 0.001^b^
Saccular (251)	155 (61)	
Elongated (32)	127 (37)	
Others (58)	157 (54)	

*ACA* anterior cerebral artery, *ICA* internal carotid artery, *MCA* middle cerebral artery, *Nav* number available, SD standard deviation

^a^ Welch’s t-test

^b^ Welch’s ANOVA

^c^ F-test of linear model

### Analysis of post-operative complication

Subdural hygroma and infection occurred in 7 cases after surgery, followed by 6 cases and 5 cases of infarction and nerve injury (Table 4). No factor showed a significant difference among all the variables (Table 5).

**Table 4:** Baseline characteristics of complication

Complications	Number of patients (% in total 420 operations)
*Short-term complication*	
Hygroma	7 (1.7)
Infection (Meningitis, wound infection, etc.)	7 (1.7)
Infarction	6 (1.4)
Nerve injury (Optic nerve injury, etc.)	5 (1.1)
Chronic subdural hematoma	3 (0.7)
Bone flap problem (Extrusion, etc.)	3 (0.7)
Wound swelling with no infection	2 (0.5)
EDH	1 (0.2)
CSF rhinorrhea	1 (0.2)
Total	35 (8.3)
*Long-term follow up*	*N/Nav (%)*
Memory loss	37/396 (9.3)
Olfactory dysfunction	66/380 (17.4)
Scalp sensory change	99/378 (26.2)

*CSF* cerebrospinal fluid, *EDH* epidural hemorrhage, *Nav* number available

**Table 5:** Analysis of post-operative complications

Variable (Nav)	Post-operation. complications (%)	P-value
Total (420)	35 (8.3)	
Age (420)	35 (8.3)	0.061^a^
*Sex*		1.000^b^
Male (138)	12 (8.7)	
Female (282)	23 (8.2)	
*Rupture*		0.883^b^
Unruptured (203)	16 (7.9)	
Ruptured (217)	19 (8.8)	
Hunt-hess grade		0.246^b^
Grade 0 (202)	15 (7.4)	
Grade 2 (161)	12 (7.5)	
Grade 3–(57)	8 (14)	
*Aneurysm size*	34 (8.7)	0.220^a^
*Location*		0.450^b^
ICA aneurysm (106)	7 (6.6)	
ACA aneurysm (174)	18 (10.3)	
MCA aneurysm (140)	10 (7.1)	

*ACA* anterior cerebral artery, *ICA* internal carotid artery, *MCA* middle cerebral artery, *Nav* number available

^a^ Likelihood ratio test of logistic regression model

^b^ Pearson’s chi-square test

Memory loss, olfactory dysfunction and scalp sensory change were analyzed according to the location of the aneurysm (Table 6). Memory loss and olfactory dysfunction were much more happened in ruptured aneurysm group, but scalp sensory change was more significantly happened in unruptured aneurysm group. And memory loss, olfactory dysfunction, and scalp sensation change all showed significant differences by location (p = 0.015, p < 0.001, p = 0.004).

**Table 6:** Analysis of Memory loss, Olfactory dysfuction, and Scalp sensory change

Variable	Memroy loss		Olfactory dysfunction		Scalp sensory change	
	N/Nav (%)	P-value	N/Nav (%)	P-value	N/Nav (%)	P-value
Age		0.128^a^		0.075^a^		0.250^a^
*Sex*		0.897^b^		0.493^b^		0.674^b^
Male	13/130 (10)		19/126 (15.1)		35/125 (28)	
Female	24/266 (9)		47/254 (18.5)		64/253 (25.3)	
*Rupture*		0.008^b^		0.001^b^		0.028^b^
Unruptured	10/195 (5.1)		21/192 (10.9)		61/192 (31.8)	
Ruptured	27/201 (13.4)		45/188 (23.9)		38/186 (20.4)	
*Hunt-hess grade*		0.018^b^		0.003^b^		0.076^b^
Grade 0	10/195 (5.1)		21/192 (10.9)		61/192 (31.8)	
Grade 1–2	20/150 (13.3)		33/144 (22.9)		31/142 (21.8)	
Grade 3–s	7/51 (13.7)		12/44 (27.3)		7/44 (15.9)	
*Aneurysm size*		0.084^a^		0.776^a^		0.311^a^
*Location*		0.015^c^		< 0.001^a^		0.004^a^
ICA aneurysm	4/101 (4)		19/99 (19.2)		32/97 (33)	
ACA aneurysm	23/162 (14.2)		40/151 (26.5)		47/150 (31.3)	
MCA aneurysm	10/133 (7.5)		7/130 (5.4)		20/131 (15.3)	

*ACA* anterior cerebral artery, *ICA* internal carotid artery, *MCA* middle cerebral artery, *Nav* number available

^a^ Likelihood ratio test of logistic resgression model

^b^ Pearson’s chi-square test

^c^ Fisher’s exact test

**Fig. 4:**
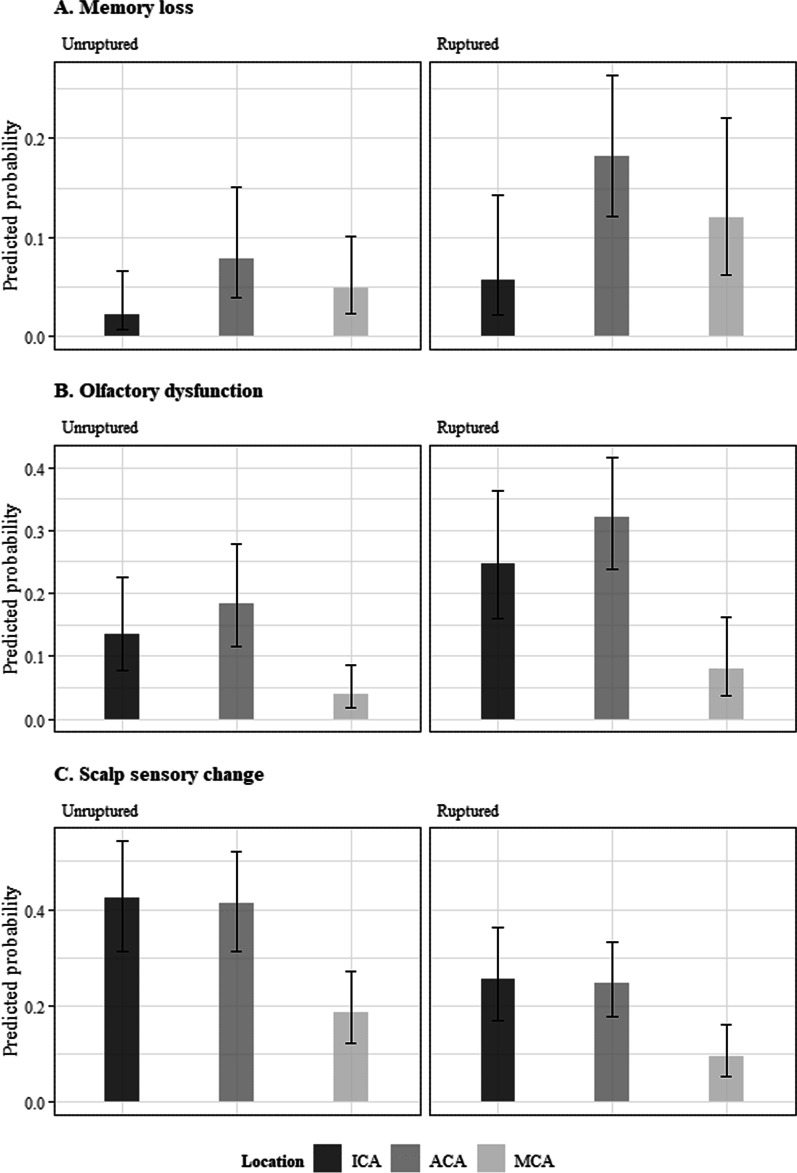
Predicted probability of Memory loss, Olfactory dysfunction, and Scalp sensory change. *ACA* anterior cerebral artery, *ICA* internal carotid artery, *MCA* middle cerebral artery

When a multivariate model was established for each event, the presence of rupture made significant affect in incidence of memory loss and olfactory dysfunction (p = 0.010, 0.010 respectively). For the scalp sensory change, ruptured status (p = 0.002) and aneurysm location (p < 0.001) were found to be significant effect (Fig. 4).

## Discussion

The authors tried to clip good grade anterior circulation aneurysms through trans-eyebrow keyhole approach. So, almost ICA and ACA aneurysm cohorts treated by supraorbital keyhole surgery. Most neurosurgeons adopt the pterional approach first for clipping of MCA aneurysms [5]. But the authors treated MCA aneurysms when keyhole surgery was feasible according to CT simulation technique which provide a shorter straight distance from the skull. If the aneurysm located more lateral and the pterional route was shorter than half the length of the supraorbital route, the pterional approach was used. The selection was based on the 3D simulation of the distance from the bony surface to the aneurysm, not the length of M1. The supraorbital approach was deemed more advantageous for dissection when M1 was short. Although there is a report on choosing the operative method based on the length of M1 [6], the authors used CT simulation to compare the distance from the bony surface to the aneurysm between the pterional approach and keyhole surgery. This method may be more practical for each patient. In most cases, the operator used a surgical micro-mirror for complete clipping. The surgical mirror or endoscopic assistance was helpful in clipping the aneurysm completely due to the small open corridor.

There is no significant difference between the ICA, ACA, and MCA aneurysm groups in the distribution of GCS, so it is considered sufficient data to analyze the outcome and factors analysis with ICA, ACA, and MCA aneurysm groups.

In this study, MCA aneurysm group was found to have a longer operation time than ICA and ACA aneurysm group. For the clipping of ICA or ACA aneurysms, partial sylvian dissection is needed during supraorbital approach. For the MCA aneurysm clipping, wide sylvian dissection is needed mostly, so the operation time is longer than ICA or ACA aneurysms. It was known that the longer the operation time, the more significant the complications occurred [7], but the results of this study did not correlate with the complication and operation time.

The rate of complications after conventional craniotomy in aneurysm surgery is various, but approximately, about 15–25% [8–10]. Complication rate of this study was 8.3%, which was a relatively low rate compared to conventional craniotomy. There was no difference depending on the location of cerebral aneurysms. And there was no case that needed re-operation due to epidural or subdural hemorrhage or infection. There were 3 cases of protrusion of bone flap, which needed reposition of bone flap for cosmetic purpose. Postoperative complications were checked at the outpatient department 6 months after the operation. Memory impairment and olfactory dysfunction can be transient postoperatively and gradually improve in many patients, so we evaluated the final neurological deficiencies 6 months after the operation. There were a few patients who complained of memory loss or olfactory dysfunction after 6 months, but their neurological symptoms did not improve in our experience up to a 10-year follow-up. However, patients who complained of sensory changes on the scalp showed improvement after 6 months and almost full recovery after 3 to 4 years. In ACA aneurysm group, more memory loss occurred than other locations, but no difference in the frequency of complication occurrence comparing with conventional ruptured anterior communicating artery aneurysm surgery such as hypothalamic perforator injury or hippocampal insufficiency [11, 12].

Olfactory dysfunction is also the highest incidence in ACA aneurysm group. Which is an inevitable complication because this approach needs more frontal lobe retraction and resulting a higher incidence of olfactory dysfunction than conventional approaches. To minimize this, it is useful to do olfactory nerve dissection carefully with reference of the report that the incidence of olfactory dysfunction was lowered through olfactory nerve dissection [13].

After surgery, patients become sensitive to the eyebrow incision and complain about sensory changes around the supraorbital nerve dermatome. Immediately after surgery, complaints were mostly dull dysesthesia and it changed to the abnormal sensation in about 3–6 months. During this period, the patients usually got more sensitive. The authors examined these scalp abnormalities in three stages: normal, slightly uncomfortable, and much uncomfortable, at 6 months after the surgery. In the case of complaining of abnormal feeling of discomfort up to 6 months after surgery, it was classified as a complication.

In transciliary keyhole surgery, the supraorbital nerve is a susceptible structure [14]. Sensory change occurred in 26% of patients and was less pronounced in MCA aneurysm group, despite significant efforts for preservation of the supraorbital nerve during surgical treatment. (p = 0.004) In the process of making incision and bone flap, there were more unruptured aneurysm cases in the MCA aneurysm group, and craniotomy size in the unruptured aneurysm group is smaller than in the ruptured aneurysm group, and it can explain the possibility that the supraorbital nerve was actually less damaged in the MCA aneurysm group. It is estimated that less nerve injury occurred due to less skin incision size. In our cases, the skin incision did not extend beyond the lateral margin of the eyebrows and the bone was exposed through sub-pericranial dissection, meaning under the temporalis muscle attached to the superior temporal line. Since the facial nerve is located deeper in the superficial temporal fascia, this layer was protected during bone exposure. In our series, there was no patient who had facial palsy after the operation.

**Fig. 5:**
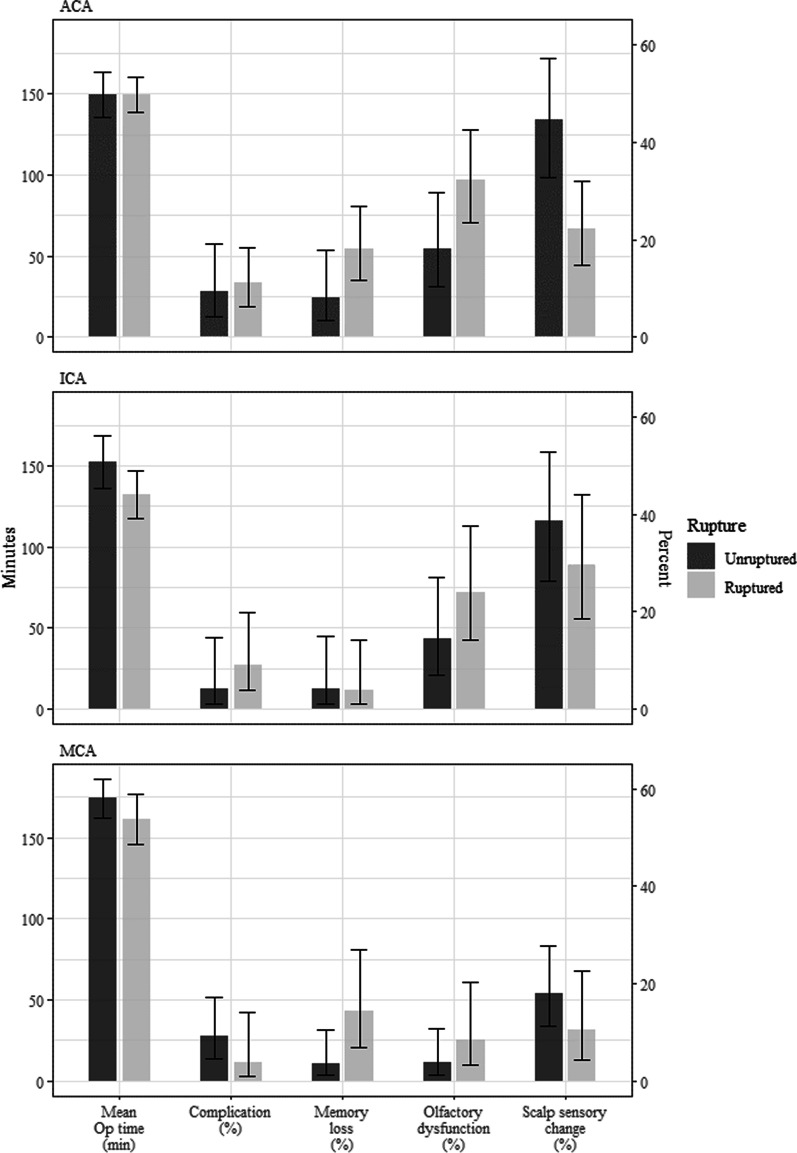
Demographic comparison of factors between ruptured and unruptured aneurysm. *ACA* anterior cerebral artery, *ICA* internal carotid artery, *MCA* middle cerebral artery

The operation time was shorter in ruptured cases (Fig. 5). In the analysis of operation time, one assumption as to why the operation time seemed to be shorter in ruptured aneurysm with high grade Hunt-Hess grade group is that ruptured aneurysm cases are usually treated urgent situation. So all the process focus in straight forward early clipping and frequent use of temporary clipping. Also there would have been a selection bias that relatively favorable case for keyhole clipping is selected in ruptured aneurysm cases. And in the case of unruptured aneurysm, even a relatively complex shape of aneurysm considers keyhole surgery preferentially, the surgical process can be a little more careful.

Both memory loss and olfactory dysfunction were found to be occurred more frequently in patients with ruptured aneurysm and the results are as same as conventional surgery. Scalp sensory change was dominant in patients with unruptured aneurysm, especially MCA aneurysm group. Since the surgical procedure is the same for ruptured aneurysms or unruptured aneurysms, it is not likely that there will be a difference due to surgery. The patients with unruptured aneurysm may responded more sensitively because some patients suspect a scalp sensory change as a signal of brain lesion. But when patients got explanation about clinical course of recovery after nerve injury, their complaints of sensory change were decreased. Therefore, in case of eyebrow incision for clipping unruptured aneurysm, it is necessary to detailed explain about the sensory changes before surgery.

Another interesting point is that although there was a large difference in the total number of patients, no post-operative complication occurred in elongated aneurysm group and 19 complications were occurred in saccular aneurysm group. Studies have shown that the probability of complication, such as infarction or intraoperative rupture (IOR), increases as the shape of the aneurysm becomes irregular [15, 16]. We suggest the possibility that because the pathophysiology of saccular aneurysm is thinning vessel wall to make the aneurysm [17, 18], in small sized aneurysm, the clip is often involving a part of the parent vessel for a complete clipping. In this situation, complications may have increased. In case of elongated aneurysms, mostly when neck is exposed, aneurysm clipping is done easily, relatively. In the same context, the operation time was also shorter in clipping of elongated shape aneurysm group.

IOR occurred in total 27 cases. 26 cases occurred after arachnoid dissection where proximal control was possible, and the surgical procedure did not differ from conventional surgery and did not affect complication rate. One case occurred before arachnoid dissection. In our case, manual compression of the craniotomy site about 5 min and bleeding was stopped, and clipping was performed after frontal lobe partial resection. The patient discharge with modified Rankin Score I.

Keyhole surgery is suitable for minimally invasive surgery for patients, but there are disadvantages for the surgeon such as difficulties in recognizing anatomy and limited instrument use. The pterional approach can provide a large corridor and clear anatomy recognition, but it can result in tissue damage due to a larger wound and increased postoperative pain. Based on patient factors and with guidance from CT simulation, keyhole surgery can be more appropriate for aneurysm operations in many cases.

### Limitation

The authors try to present the characteristics of selected cases for keyhole surgery so selection bias may occurred in the analysis of affecting factor for complications. Before the analysis, it was expected that the configuration of sylvian fissure would be an important factor in the analysis of operation time for each location, but due to the nature of transciliary keyhole surgery, there was not many cases that the configuration of sylvian fissure was clearly confirmed. There was no significant difference in our analysis, but thorough analysis of the sylvian configuration will be additionally required.

The number of patients included in the overall analysis was not small, but as a retrospective study, not all factors could be evaluated in all patients due to lack of records. There is a possibility of an error due to this point, and for this, verification through a larger scale study and a prospective study as needed is necessary.

## Conclusion

The most suitable location for the eyebrow incision approach is ICA, ACA aneurysm. Unruptured MCA aneurysm can be operated with this approach, but minipterional approaches were more performed in the case of ruptured MCA aneurysms. MCA aneurysm group had longer operation time than ICA and ACA aneurysm group, but there was no significant difference in complication. Memory loss and olfactory dysfunction were more found in ACA aneurysm group. On the other hand, in the overall comparison of ruptured aneurysm and unruptured aneurysm group, it was confirmed that ruptured aneurysm cases can show good results with a keyhole approach if selected well.

## Data Availability

The datasets used and/or analysed during the current study are available from the corresponding author on reasonable request.

## Abbreviations

ACA: Anterior cerebral artery
CSF: Cerebrospinal fluid
CT: Computed tomography
GCS: Glasgow coma scale
ICA: Internal carotid artery
ICH: Intracerebral hemorrhage
IOR: Intraoperative rupture
IQR: Interquartile range
MCA: Middle cerebral artery
QOL: Quality of life
3D: Three dimensional
